# Making sense of COVID-19: manifestations of health capital during the pandemic

**DOI:** 10.1186/s12889-024-18451-8

**Published:** 2024-04-01

**Authors:** Ş.Erhan Bağcı, Şengül Erden, Begüm Yengel

**Affiliations:** 1 Department of Medical Education, Faculty of Medicine, Çukurova University, Adana, Turkey; 2 Medical Education, Gazi University, Ankara, Turkey; 3 Department of Medical Education and Informatics, Faculty of Medicine, Ankara University, Ankara, Turkey; 4 Department of Foreign Languages, Middle East Technical University, Ankara, Turkey

**Keywords:** Health capital, Habitus, Field, COVID-19, Social determinants of health, Documentary method, Biographical narrative interview

## Abstract

**Background:**

Grounded in Bourdieu's theory of human practice, this study aims to examine how individuals as social agents made sense of and acted upon their COVID-19 experiences. A recent conceptualization of health capital is utilized to explain the practices of patients in the pandemic, in relation to their biographical background.

**Methods:**

This is a qualitative research in which the data were collected by biographical narrative interviews through a theoretical sampling approach. Eighteen interviews with COVID-19 patients were conducted and 8 of them were analyzed by the Documentary Method.

**Results:**

The informants made sense of their illness experiences through their health capital, which is manifested in their self-perception of health, their attitudes towards the healthcare system, their conception of terms such as luck, their work status, and the gendered division of labour at home in the COVID-19 pandemic. All the manifestations are mediated by the social, cultural, and economic capital of the informants, and their habitual practices are based on their symbolic capital.

**Conclusion:**

The study depicts how social agents’ health capital manifested in the pandemic, relying on their symbolic capital, and shaping their practices. Further research across diverse contexts is needed to fully understand extra dimensions of health capital as a descriptor of the social determinants of health.

## Introduction

COVID-19 has been a severe public health issue, which caused a drastic and long-lasting shock on a global scale. It not only jeopardized individuals by exposing them to a life-threatening illness but also endangered their very existence by radically altering their ordinary lives [1–3]. Therefore, COVID-19 can extend beyond individuals' health, encompassing their living conditions.

Even though the source of the threat was the same for everyone, the impact of the pandemic varied for different social groups. The World Health Organization [4] states that societies are comprised of inequalities in terms of income and social protection, education, unemployment, job insecurity, working life conditions, access to affordable health services of decent quality, and the like, which result in differences in their health status. Health is always determined by the conditions of the population in the individual lives [5]. Relatedly, studies conducted on the COVID-19 pandemic show that the social conditions in which people are born, grow, work, and live significantly determine their vulnerability to COVID-19. Mortality among minority groups because of COVID-19 is found to be greater than the rest of the population because of their economic disadvantage and limited access to quality medical care [6, 7]. Particularly in low-and-middle-income countries, women, households with children, and the ones with the lowest socioeconomic status are more vulnerable before COVID-19 [8]. Social class-based inequalities are also found to be significant in the pandemic [9]. These studies with many others [see 10–13] highlight that COVID-19 has been more than a clinical case with significant social dimensions. Therefore, in addition to the disease itself, and the quality of medical care a person receives, it is important to recognize the pivotal role of social factors in the overall experiences of illness.

The aforementioned studies on the social dimensions of the pandemic focus on the social determinants of the disease, particularly on the social reasons that made specific groups of any population ill. However, there is a dearth of research on what the patients experienced after their COVID-19 diagnosis. This study aims to examine how individuals as social agents made sense and acted upon their COVID-19 experiences, in relation to their biographical background. It is expected that understanding the experiences of those diagnosed with COVID-19 will help to guide the overall measures against any future outbreaks.

### Theoretical framework

It is not experiences that befall us, but events. Experiences are constructed through the interpretive frames we employ to assign meaning to events [14]. It is the individuals, more precisely social agents, who make sense of their experiences out of events by fundamentally shaping how they experience these events. Therefore, gaining an insight into how social agents make sense of their experiences requires a comprehensive understanding of how they construct them.

Stating that it is the social agents who construct and make sense of experiences underlines that human practices as results of these experiences do not happen incidentally. Bourdieu and Wacquant [15] state that human beings are social agents that are products of the history of their accumulated experience of a path within the whole social field, which means they do not act solely on internal motives. On the other hand, they are not totally determined by their life-world but act in relation to it. Thus, in order to understand the COVID-19 experiences of social agents, there exists a need for a set of concepts that would allow situating human practices within social and historical conditions.

Bourdieu employs the terms habitus, field, and capital to explicate human practice. Habitus provides a thinking tool for considering practice and knowledge interdependently with the social milieu in which it is generated [16]. It is the system of mostly durable but transposable dispositions; the structured structures predisposed to function as structuring structures [17]. It refers to a practical rationality inherent within a specific historical system of social relations that is transcendent to the individual. One can consider habitus as the socialized subjectivity constructed through the biographical experience of social agents. It serves as a guiding principle to cope with diverse circumstances effectively allowing social agents to strategize within their social field [15].

The field is simply the social structure in which habitus is generated. It enables comprehending the social structures and practical actions of the social agents in them, relationally. Bourdieu [15] states that there is a correspondence between the field and social agents’ mental structures, determining how individuals perceive and categorize the social world. Social agents make sense of their experiences through these mental structures and act upon them, while concurrently reconstructing their habitus that would guide subsequent actions.

The field is not a vacuum; rather, habitus is constructed among various asymmetric forces in the field that confront each other permanently. What enables the social agent to hold an appropriate position in this field of struggles is the possession of social, economic, and cultural capital [15–18]. Bourdieu [19] elaborates on the notion of capital as any resource effective in a given social arena:

… economic capital … is immediately and directly convertible into money and may be institutionalized in the form of property rights; … cultural capital … is convertible, in certain conditions, into economic capital and may be institutionalized in the form of educational qualifications; and … social capital … [is] made up of social obligations (“connections”), which is convertible, in certain conditions, into economic capital and may be institutionalized in the form of a title of nobility.

Positions within any field are distributed among social agents according to the overall volume and the composition of their symbolic capital (social, economic, and cultural) and the relative weight of that regarding the total assets in the whole field. Briefly, habitus generates practices, beliefs, perceptions, and the like, based on its symbolic capital within the field, influencing one's position in relation to other social agents who are also striving for better positions. Therefore, notions of field, habitus, and capital can be employed to understand the attitudes and behaviours of patients, if one needs to discover their experiences of illness. This study draws on the theoretical framework of Bourdieu, by moving forward to the concept of “health capital”, which implies the biographical resources of any actor that enables them to act in cases of health or illness in a given field.

Grossman [20] developed a model of the demand for good health as a commodity in which he coined the term health capital. In his work, health is regarded as “a durable capital stock that produces an output of healthy time” [20]. His view of health capital seems to be rooted in solely economic principles whereas Shim [21] proposes the term, “cultural health capital”, with which she refers to the repertoire of cultural skills, verbal and nonverbal competencies, attitudes and behaviors, and interactional styles, cultivated by patients and physicians. She asserts that the concept helps examine patient-physician relationships to overcome the relational dynamics of unequal treatment [22, 23]. On the other hand, in their research on the relationship between health capital and occupational status, Harsløf et al. [24] introduce a different definition which refers to conspicuous health consumption and the display of virtuous health-related practices which includes both action and inaction. From a more comprehensive perspective, Schneider‑Kamp [25] states that health capital encompasses the field-dependent skills, competencies, social relationships, financial means, and status that can be employed toward the preservation of good health and the management of illness, immediately or mediated through conversion from other forms of capital.

This study which focuses on the illness experiences of COVID-19 patients leans on the conceptualization of Schneider-Kamp’s [25], considering that the health capital of any social agent in a given field shapes and manifests their health-related habitual practices, actions or inactions, possibly by converting assets of other forms of capital. Health capital explains how a social agent makes sense, and acts or not in that very actual way in a given health-related circumstance which is the COVID-19 pandemic in this study.

### Methodology

This research which aims to reveal how the COVID-19 patients made sense of their illness experiences was conducted by the Documentary Method, which is a reconstructive qualitative methodology [26, 27], as it provides the opportunity to relate individual experiences to the social context. The data were collected by biographical narrative interviews through a theoretical sampling approach. Theoretical sampling is the process of data collection for generating theory whereby the analyst jointly collects, codes, and analyzes their data and decides what data to collect next and where to find them, in order to develop their theory as it emerges [28]. In the theoretical sampling strategy, the initial informants are selected upon a preconceived general theory, and as the interpretation of initial data develops, the subsequent informants are determined depending on the emerging theory. In this study, the initial informants were picked randomly, in order to have a general understanding of the pandemic experiences of COVID-19 patients. After the initial analysis, the severity of the disease was revealed to be a significant factor in patients’ experiences, and thus new informants were picked from the ones who were hospitalized for COVID-19. By further analysis, work status was also found to be effective in patients’ perceptions of the disease, and we diversified the study group in terms of the level of precarity of their working positions. New informants were found by a snowball approach. As a result, we conducted a total of 18 biographical narrative interviews, but utilized eight of them in our analysis due to their provision of the most extensive and richest data for the Documentary Analysis (see Table 1). The average duration of the interviews was approximately 43 min, whereas the average of the analyzed ones (*n* = 8) was 54 min. Each interview was transcribed verbatim and the identities of the informants were anonymized. The analysis was done by the Documentary Method which enables the researcher to develop a systematic understanding of the structure of meaning beyond the subjectively intended meaning of the actors while retaining an empirical and analytical focus on the knowledge of the actors themselves [29].

**Table 1:**
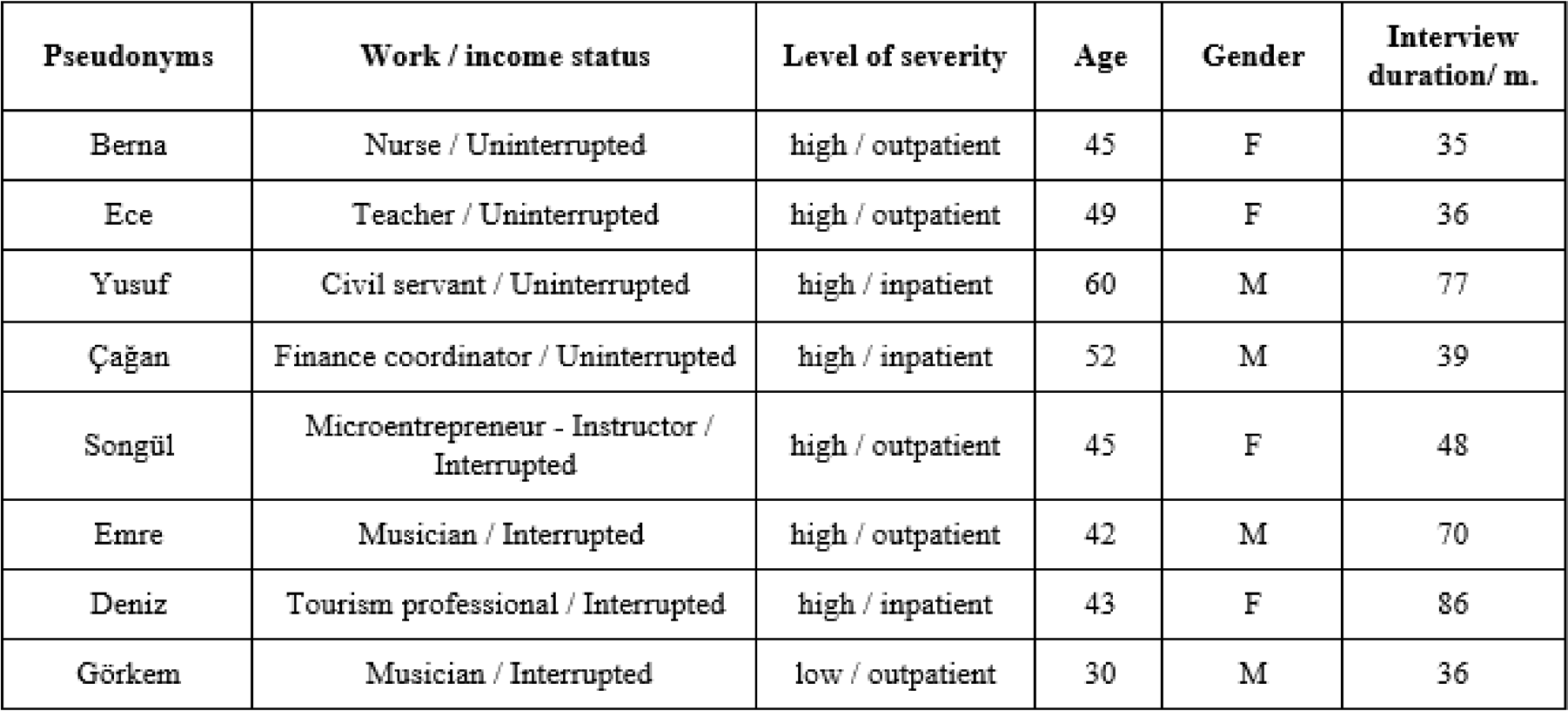
Informants of the study

Ethical approval was obtained from the [blinded for peer review] (approval no. [blinded for peer review]). All informants provided written informed consent before enrollment in the study.

## Results

The analysis of the biographical narratives of the informants in this study revealed five themes that provide an understanding of how the informants made sense of and acted upon COVID-19, which are their self-perception of health, (mis)trust in the healthcare system, perceived role of luck, their work status, and gender.

### Self-perception of health

Documentary analyses of the narratives of the informants in this study revealed that the primary building block of one’s health capital is the self-perception of health. In other words, an informant’s self-perception of health seems to play a crucial role in determining their conduct during the pandemic. Ece is a teacher who was hospitalized due to severe COVID-19:

… I had frequent herpes outbreaks in my childhood. I frequently had them in my childhood, but they lessened as I grew up, I think of it… I am prone to getting sick often. I had to have a hysterectomy six years ago, I experienced early menopause … I think there are more diseases like this in my life …

Ece depicts herself as an individual with a history of recurring health issues throughout her whole narrative. She mentions several times that she gets ill quite often. In earlier phases of the pandemic, she immediately sought medical consultation upon experiencing a cough. Nevertheless, she was not diagnosed with COVID-19. She was so sensitive about the measures that she wore double masks at school. Therefore, she was not expecting to contract it when she was diagnosed with COVID-19.

Yusuf, on the other hand, who had to be treated at the intensive care unit for COVID-19, was not expecting to catch the disease, not because he followed the measures carefully, but because he considers himself healthy. He works as a public servant, and he was at work at the onset of the first symptoms:

My nose started running and I got a severe headache on the sides of my head … After that, a slight cough, not a lot, after that, I said to my friends at work, I said to my colleagues at work, I had a headache, I would never have a headache, I said to my friends, after that, anyway. I'll take a leave so that I can go and rest a bit; that day I came home and rested. Then I got up in the morning again, I was a little tired, my reflexes were slow and I went back to work again...

Amidst the pandemic, he has the symptoms, but he still does not think he is ill because he has quite a strong and healthy self-perception. This is why he takes the day off with the first symptoms but gets back to work the day after. He mentions that he took a leave the second day just because his colleagues wanted him to do so. However, he has not yet undergone a COVID-19 test since he believes he is not ill.

Informants’ attitudes towards pandemic measures and their conduct on the first symptoms are rooted in their self-perception of health and manifest their health capital. This is generated through their biographical experiences. Ece, who perceives herself as a person prone to illness, pays more attention to pandemic measures and goes to see a doctor immediately after the first symptoms, whereas Yusuf does not even need to get tested although his symptoms were easily identifiable.

### (Mis)trust in the healthcare system

Attitudes towards the healthcare system are found to be another manifestation of health capital. Yusuf’s narrative documents that he did not trust medical staff before the pandemic until he dialed 112 for help when his oxygen saturation level dropped significantly below the normal range:

… then we called 112. The nurses arrived and I thought they were going to treat us like we had the plague. It used to be like that, they would probably stand away from us, I thought or that is what we heard and saw. After that, they asked me a question in a very sincere way, I don't know what they did, they took me and left. It turns out that they were vaccinated, so the thing is not easily transmitted to the vaccinated person. I used to think there would be no benefits of the vaccine. Then they brought me to the hospital in an ambulance.

He had anticipated the medical staff to distance themselves from him and display a negative attitude when they came home. However, he was pleasantly surprised to see that they were very kind and supportive towards him. He assumes that their ease and willingness to assist may have been because they were vaccinated. He mentions that he had not comprehended the advantages of getting vaccinated before. He remarks his first encounter with the medical staff from the emergency service as a moment of transformation, which later on made him speak about the nurses at the hospital empathetically. He describes the chaotic circumstances there and appreciates the effort of the medical staff although he emphasizes that he was left unattended for a long time at the service.

However, even though his beliefs transformed, he still maintains a degree of skepticism towards the healthcare system. When he needed reliable information about his health status, he opted to consult with one of his relatives who is a physician residing in a different city, by phone, which he mentions: “I have a nephew, a doctor, he got information from my doctors and something from time to time, then he called me and told me to stay calm.”

In his narrative, Yusuf recounts that he consulted his physician relative, not the ones at the hospital, for information about his health on multiple occasions, both prior to and following his hospitalization. He employs his social capital as part of his health capital several times when he was ill. This type of conversion is also significant in Deniz’s narrative:

In the meantime, my husband called someone we know because I wouldn't be able to complete this process at home, it just was not possible. But when you go there, they don't want to admit you as an inpatient. They'd rather send you home. It wasn't a pandemic hospital, but, of course, there was the COVID department and inpatients... They carried out my hospital procedures with the help of the folks we call...

She says that her condition was worsening and her first admission to get hospitalized was denied because it was not a special hospital for COVID-19. Her husband had to call their friends to get her hospitalized, which means they managed to have a bed at the hospital by employing their social capital as part of their health capital.

What lies under the informants’ lack of confidence in the healthcare system is their unfavorable biographical experiences, which seem to be a significant dimension of their health capital. Findings highlight that positive experiences might alter this attitude to a certain extent. However, it is not easy to transform this kind of perception because habitus is deeply rooted in its biography. This is why the informants in the study still rely on their social capital rather than the healthcare system to address their health concerns.

### “Luck” as a means of sense-making

Another dimension of the informants’ health capital in this study is that they apply to a discourse of luck to explain both their treatment and recovery. In other words, they attribute their health status to luck, not to medical intervention. Çağan who works at a factory as a finance coordinator was hospitalized at the intensive care unit due to severe COVID-19. He identifies some critical moments in his narrative, in which his luck took a turn:

There was a great chance that there were available beds at the hospital at that time because, towards the end of that December, there was a decrease in COVID in Turkey… Miraculously, when I got admitted as an inpatient on Saturday, there was a need for immunosuppressants, and they had them, thank God.

Çağan considered himself healthy before the disease, but he got COVID-19 unexpectedly. At first, he was not hospitalized although he experienced a significant decline in his health, but after a short while a physician called him, and they found a bed for him. Then, he received treatment with an immunosuppressive that was rarely found. He does not say that the medical system worked and helped him recover, but does emphasize that he was lucky.

It is noteworthy that in most of the informants' narratives in this research, they utilize terms such as luck, chance, fate, and fortune to explain both their treatment and recovery. While they are aware that they survived due to their good health or to the effectiveness of prescribed medications, they apply to these terms as interpretive tools for expressing their experience. It reflects an interplay between their cultural and health capital, which can be associated with the skepticism towards the healthcare system that was mentioned in the previous section as well.

### Work status matters

The work status of the informants, coupled with their symbolic capital, emerges as another crucial aspect of health capital in understanding how habitus functions during a pandemic. When speaking of COVID-19, informants who have a secured work status, such as a nurse, a teacher, or a public servant at a state institution, focus on health considerations with almost no word on their job, whereas the others who work at precarious positions prioritize work-related challenges caused by the pandemic in their narratives. Answering the question about the pandemic, Deniz, who works at a tourism agency, primarily mentions the issues in her sector:

…speaking of the pandemic, we all entered uncharted territory after March 2020. As a matter of fact, as a sector, ours, the tourism sector, is affected very quickly by all kinds of things, unfortunately... When the pandemic started, of course, we also followed it on social media on television; we always followed very closely what was going on, and as part of our job, we entered into an obscurity...

The pandemic had a substantial impact on numerous economic sectors, including the one in which Deniz worked. Despite having been hospitalized due to severe COVID-19, Deniz primarily emphasizes the challenges confronted by the tourism sector. This suggests that these challenges were a key determinant influencing her pandemic experience. Her narrative documents that working conditions are of primary importance in the pandemic experiences of individuals as social agents.

It was similar for Emre, who severely suffered from COVID-19 at home. He was working as a musician at a restaurant at the onset of the pandemic. That the entertainment sector was one of the first to get affected by pandemic measures draws the framework of his whole narrative, through which he depicts his pandemic experience as almost an economic issue:

The coronavirus hit my sectors directly. First, the restaurant and then the stage closed… First, the music ended, and the restaurant I worked in was also closed. I mean, the restaurant where I worked as a musician… I was there, but the operations continued in this restaurant because it gave food to nursing homes… Therefore, I could have a work permit. However, I was fired, in other words, I was sent on unpaid leave… It is something like being fired. All of a sudden, the sector collapsed. There is no music; restaurants are closed…

The pandemic disrupted his professional life since the restaurant at which he was employed halted operations. He had to take an unpaid leave because although the restaurants continued working with delivery services, there was no room left for musicians like Emre. His wife was still working, but he was bored and impecunious at home, so he asked the restaurant management to employ him as a deliveryperson, and they accepted it. He notes that despite the insufficient compensation, he persisted in his employment there as he lacked alternatives. He had a significant loss of income and status, but he was not able to find another solution to survive.

Unlike Emre, Görkem, another musician who recovered from COVID-19 with relative ease, managed to formulate a strategy that assisted in his progress surpassing his prior state. He mentions that he was depressed at the onset because the venues where he performed were all closed and all his contracts were canceled. Then some of his friends invited him to produce music videos for online distribution at home:

… later on, I was included in some of my friends' videos, moreover, on social media, the content they produced started to become very popular, including me… Yes, I had a friend who was engaged in this business, he included me in his videos and I included my friends, then these videos spread a lot; job opportunities arose for us from social media. Also, everyone is locked in their homes and cannot go out. All musicians have lost their job opportunities. Of course, interaction has increased in social media. It is because people are at home spending time with their cell phones and computers. We decided to turn the band into a project, then we created a page and started uploading the videos we shot there; then this business grew so much that one day (a famous singer) contacted us. Then we started playing with her, and we started performing with her. Then we started to operate in corporate areas with our project. I didn't know beforehand that there was such a market.

While seeking survival, Görkem found a new music performance platform that he would not have known if it was not for the pandemic. He says that he and his friends in close proximity assembled to shoot videos or conduct live performances online. It seems that Görkem transformed his social and cultural capital into economic capital successfully.

Online survival strategies were on the move throughout the pandemic. Songül also suffered from COVID-19 severely at home. Similar to Görkem, she was able to employ a successful work-related online survival strategy in the pandemic, despite her precarious work status. She had been running a small business in textile manufacture and also working as an instructor at a Public Education Centre before the pandemic. Both were closed with the outbreak:

We had a lot of trouble during the pandemic and my workplace was closed, I mean it had to close, more precisely, our workplaces were closed… I started to get my textile orders over the phone and started showing the models on the Internet… At that time, I tried to do things from home; I had payments I had to take care of. I got a few orders that we tried to get by with, but my husband had no job, we tried to manage anyway until we got over the pandemic.

Songül mentions that her husband was already unemployed, and it was she who had to come up with a solution. She developed a novel strategy for her work by carrying her business into an online setting through which she presented textile models to her clients. She says she had been able to use the Internet already before the pandemic but had not considered the application of online resources to her job. However, because of the lockdowns and other pandemic measures, she had to find a solution to continue working and immediately decided to move her job to the online platform. Although it was not as effective as before, it helped her get by. She transformed her cultural capital, namely her ability to use the Internet, into economic capital.

Work status, coupled with symbolic capital, was revealed as a noteworthy dimension of health capital when examining how social agents’ habitus reacted during the pandemic. Compared to those in secured positions, the pandemic appears to be primarily an economic issue for precarious workers, as it has put their previous income and status at risk. The ones who could not develop a successful survival work strategy encountered a significant loss in their income and status. On the other hand, the analysis reveals that some of the informants leveraged their symbolic capital to navigate the evolving unfavorable circumstances subtly, transforming other types of capital into economic capital. The interplay between the work status and the symbolic capital was a significant manifestation of health capital during the pandemic.

### Home: safe yet gendered

During the pandemic, people took measures to isolate themselves from others to avoid contracting the disease. These measures involved wearing masks and maintaining physical distance in public spaces, but the primary means of avoiding the disease was staying at home to the fullest extent possible. Analyses of the narratives of the informants in this study indicate that they perceived their homes to be a sanctuary where they could find protection from the disease, and also a place of shelter for themselves if they got infected. Çağan was in another city when he tested positive:

… the test came back positive. Since I also had a fever, they said okay, you will stay at home in quarantine. Of course, but I am in Istanbul, I am alone. I had a difficult night on Wednesday and the next morning I said, “It can’t go on like this, I’m alone here, and if something happens to me, no one will know of it”. I said in a final attempt, “at least I’ll go to Ankara”. I got up on Friday morning at 10 o’clock and jumped in the car. I came straight to Ankara… here my wife had already prepared a place for me in another room because she knew it; I went to bed there.

When he tested positive in a different city, he experienced a sense of loneliness and apprehension. He subsequently decided to drive back home, where he relied on his wife for care and support. He was uncertain about himself, and he sought comfort in the familiar and secure environment of his own home. His narrative documents the restricted nature of supportive relationships during the pandemic, which has largely been limited to one’s immediate household members. For that reason, one can claim that home emerged as the predominant site of solidarity amidst the pandemic.

Household routines were rearranged to address healthcare requirements, such as caring for the sick and safeguarding against the transmission of the virus. Berna’s narrative brings forward her worry about transmitting the disease to her household members. She is a nurse and she had a severe COVID-19:

I am in a separate room. You know, my husband is going in and out. My son, 9-year-old, has epilepsy. Well, we were afraid that he would get infected, and what if it triggered his epilepsy. So, let’s test them too, you know, at least we take the child away. This time the child is sitting in front of the door crying; he wants to be in touch.

She speaks of her concern about transmitting the disease to her son, and how it was difficult to keep him outside the room in which she had isolated herself. The main point of the arrangements at home was to ensure the safety and the survival of the household members. However, it is noteworthy in this study that rearranging household routines against the disease was especially more significant in the narratives of female informants. They give details by emphasizing their concern for the health of the households. Deniz mentions her first reaction to her positive test result:

I got informed that day in the afternoon. It was half past six. We were eating; even though I stayed away, we were in the same room, we were in the living room with my daughter and my husband. I got up right away, of course, I immediately went to the bedroom, don’t come, let me get isolated.

Just as she heard that she tested positive, she says she immediately isolated herself at home, like Berna. After that, her husband got ill and their household routine turned into chaos because there was nobody to take care of their daughter. They isolated themselves from each other at home, but when both she and her husband got hospitalized, they had to leave their daughter alone at home. Fortunately, Deniz’s sister visited her every day, but still, she was very concerned about her.

Findings suggest that the social actors regarded their homes as safe havens where they could exclusively access the care required when they contracted COVID-19. Within this context, immediate family members served as the primary source of support, and the home emerged as the central site of solidarity during the pandemic. Household routines were adjusted to meet healthcare requirements and ensure the well-being of all residents. Notably, the study highlights the greater commitment of female informants to these domestic arrangements, shedding light on the gendered dynamics within household relationships.

## Discussion

COVID-19 was not only a clinical issue but also a social one. This research seeks to uncover how social actors made sense of their COVID-19 experiences and acted upon it, or in other words how the pandemic affected their habitual practices, by applying to a recent conceptualization of, “health capital”, inspired by Bourdieu’s theory of practice, which is utilized to gain insight into the factors that drive social agents to or deter them from particular behaviors and decisions during the ongoing pandemic.

The self-perception of health held by the informants in this study is a crucial component of health capital, and any social actor’s own biographical experience of illness, regarding whether they had experienced frequent or infrequent illness, is a significant determinant of it. In a similar vein, social actors’ level of trust in the healthcare system derived from their past experiences appeared to be a manifestation of their health capital. These are significant in the analysis of the narratives since they seem to have led the informants to or prevented them from attending healthcare services for assistance. What leads individuals to the decision of whether or not to seek medical attention when experiencing symptoms is not only the external factors, so-called objective conditions, or the autonomous internal motives, so-called agency, but their socialized subjectivity accumulated through their biographical experiences, so-called habitus. Habitual actions of social agents are realized within the relationship of the external and the internal in a historical context [15]. This aligns with the findings of the study by Roshchina et al. [30] which emphasize that the decision to get vaccinated depends on age, family composition, education, type of settlement, employment, self-rated health, previous COVID-19 experience, and self-perceived risk of being infected. The authors state that vaccination decisions are not arbitrary, but related to the individual’s sense of self, which is constructed through experiences in social contexts. Similarly, Schneider-Kamp [31] situates the health conduct of individuals at the nexus of agency and health capital in her study on vaccine hesitancy. She describes the agency as the individual autonomous action guided by risk factors, self-efficacy, and self-management while referring to health capital as the aggregate of the actual or potential economic, social, and cultural resources possessed by a given agent that can affect the position of agents in the social field of health [31]. In general, this study underlines that one’s decision to seek medical help is an outcome of their medical background which affects their sense of the likelihood of getting sick or remaining healthy, and their level of trust in the healthcare system.

However, this attitude is altered by new experiences of encountering favorable situations, which is documented in this study. Mezirow [32] points out that any experience that is opposite to our biographical repertoire might lead to a change in our habits of mind when it opens the way to reflect critically upon our misconception. This kind of transformation might be cumulative that takes time, or epochal to happen suddenly. He emphasizes that epochal transformations are less common and more difficult. As a matter of fact, habitus is endlessly transformed: every time it is confronted with objective conditions similar to those in which it is generated, it is perfectly adapted to the field out of purpose [15] but when there is disagreement between the field and the habitus, the latter has to modify itself due to the subsequent structure. However, habitus tends to resist perpetuating structures corresponding to its conditions of production, but still, it is not necessarily adapted to its situation nor necessarily coherent; it has different degrees of integration that may or may not lead to change [33]. When the harmony between the field and the habitus is interrupted, the habitus is forced to accord, but only within the limits of the symbolic capital. Along the same line, the informants’ experiences with the medical staff and hospitals were transformative in the study, but when they sought information about their health status, or resolve any problem with the hospital, they still chose to turn to their social networks, or in other words to employ their social capital as part of their health capital, rather than relying on the healthcare system to address their concerns. That the informants in this study appear to have retained some level of mistrust towards the healthcare system, despite their points of view having transformed to a certain extent, verifies that habitus is open to change, but somehow still resilient.

Another manifestation of social actors’ health capital is the significance they attach to concepts such as luck, chance, or fate. The current study shows that the informants often use luck to interpret their experiences of getting treated or recovering. Despite being aware that their treatment or recovery was likely due to their good health or appropriate medication, they still attribute it to luck, chance, or fate. Hidano et al. [34] assert that luck perception in terms of health issues has consequences on preventive practices that result in a higher number of social contacts during the pandemic. Their study supports our finding that the discourse of luck is a significant dimension of health capital since it manifests a particular means of sense-making and conduct.

Employing a discourse of luck in terms of health issues might be considered as the interplay between cultural capital and health capital: our beliefs are products of our biographies, and shape the ways we construe the situations we encounter. However, the belief in luck as the reason for recovery can also be related to the mistrust towards the healthcare system as stated above. Bauman [35] states that luck is a gambling term as it is the very opposite of certainty. He underlines that it presumes an essentially uncertain setting, immune and insensitive to intentions and undertakings. It implies anything may happen yet the consequence of no undertaking can be reliably predicted. He attaches the ascend of the discourse of luck in the recent half-century to his notion of liquid modernity, which remarks uncertainty due to the rapid change of social life, elimination of social policies that had provided an umbrella of social and economic safety to welfare societies, and the rise of precarious ways of living. He claims that the sense of insecurity is a significant pillar of liquid modernity, and this is why people give more credit to the discourse of luck than their trust in the system and public institutions. That the COVID-19 pandemic established a climate of unpredictability in addition to Bauman’s liquid modernity might be the reason that the informants in this study applied to notions of luck, chance, or fate in their narratives, possibly related to their mistrust to the healthcare system. However, belief systems cannot be reduced to a single factor for understanding, and therefore, this contradictory nature of health capital needs a more profound investigation.

One can easily claim that the way social actors handle the issue of the pandemic is first and foremost determined by how it disrupted their lives. In light of the informants’ narratives, it appears that the pandemic was not only related to health issues but also to work-related challenges for the ones who did not have a secured income. What sets apart these narratives from the rest of the informants in this study is that they interpret the pandemic primarily through an economic lens, as they focus predominantly on the work-related issues they encountered. The health capital of these informants was economically determined. As a result of the pandemic, certain work sectors were disrupted, and the character of the work environment transformed, leading to substantial financial and social setbacks for individuals employed in these sectors. Although some of them managed to retain their jobs, the contraction of economic activity within their sectors caused great concerns, which contributed as the most significant determinant of their illness experience.

It was the informants’ symbolic capital, coupled with their work status, manifested as their health capital in the pandemic. Disruption was more unsettling for the ones who lost their jobs immediately at the onset of the pandemic. The economic determinants of these informants’ health capital were evident, but it was not only their economic capital but also their cultural and social capital that determined their practices during the pandemic. Some informants experienced a loss of income and status when unable to adapt. Conversely, others utilized their symbolic capital to navigate challenges adeptly, converting their symbolic capital into economic capital. The interplay between work status and symbolic capital emerged as a significant factor influencing health capital during the pandemic. Sennett [36] claims that those who prosper under temporary working conditions have a higher tolerance for ambiguity. He observed that in uncertain environments people need to be proactive when faced with ill-defined circumstances. In the context of the current study, the ability of any social actor to either settle for a lower position or strive for a higher one was heavily influenced by their possession of adequate cultural and social capital, and therefore the ones who acted to prosper, or in Sennett’s words the ones to had higher tolerance, were the ones who had appropriate symbolic capital to respond to the ambiguous pandemic conditions.

Practices to keep household members safe during the pandemic are found to be a significant manifestation of health capital. It was a time when solidarity was mostly confined to household members, and home was the primary space it was conducted. Findings put that home was not only the place that public pandemic measures legally forced people to stay in but also the safe sanctuary where people voluntarily sheltered when they got infected. Life at home was rearranged for the treatment and the protection of the households. Therefore, one can claim that the rearrangement of the household routine was a form of solidarity amidst the pandemic, and the analysis of the narratives in this study reveals that it was gendered. It is a considerable finding of this study that female informants speak of these rearrangements and measures to protect the households in greater detail, whereas the male participants either mentioned these less or not at all. Instead, they focus more on sharing their personal experience of being sick. It does not imply that men were negligent in any way, but that women attached more importance to the protective arrangements at home. This complies with the relevant literature on COVID-19. In their study on gender differences in COVID-19 attitudes and behaviors from eight OECD countries, Galasso et al. [37] show that women are more likely to perceive COVID-19 as a very serious health problem, to agree with restraining public policy measures, and to comply with them. Similar studies have also found that compared to men, women experienced an increased psychological vulnerability [38], and assumed a greater share of the household chores [39] during the COVID-19 pandemic. In addition, women with children have been found to experience a higher level of anxiety than those without children [40], and struggle to better fit the ideal motherhood standards of society while working from home [41]. These findings highlight the existence of the gender dimension that health capital functioned throughout the pandemic.

## Conclusion

This study suggests that social agents make sense of their illness experiences through their health capital, and the interplay between their economic, cultural, and social capital has a significant impact on it. Health capital, as the accumulated resources underlying health-related habitual practices, is constructed through the biographical experiences of social agents as documented in this study. It manifested in the informants’ self-perception of health, their attitudes towards the healthcare system, their conception of terms such as luck, their work status, and the gendered division of labour at home, in the COVID-19 pandemic. By examining social actors’ biographical experiences, this study sheds light on a specific pathway to understanding the social determinants of health although it is still too limited to grasp the whole range of possible dimensions of health capital. More studies that include diverse contexts, such as different geographies, countries, or cultures are required.

## Data Availability

No datasets were generated or analysed during the current study.

## References

[1] Andreescu FC (2021). A meditation on Covid-19 social trauma. J Cult Res.

[2] Stanley BL, Zanin CA, Avalos BL, Tracy SJ, Town S (2021). Collective Emotion During Collective Trauma: A Metaphor Analysis of the COVID-19 Pandemic. Qualitative Health Research.

[3] World Health Organization. Coronavirus disease (COVID-19) pandemic - situation reports. https://www.who.int/emergencies/diseases/novel-coronavirus-2019/situation-reports (2023). Accessed 28 Sept 2023.

[4] World Health Organization. Social determination of health. https://www.who.int/health-topics/social-determinants-of-health#tab=tab_1 (2023). Accessed 17 Oct 2023.

[5] Schofield T (2007). Health inequity and its social determinants: A sociological commentary. Health Sociol Rev.

[6] Badalov E, Blackler L, Scharf AE, Matsoukas K, Chawla S, Voigt LP, Kuflik A (2022). COVID-19 double jeopardy: The overwhelming impact of the social determinants of health. Int J Equity Health.

[7] Nöstlinger C, Van Landeghem E, Vanhamel J, Rotsaert A, Manirankunda L, Ddungu C, Meudec M (2022). COVID-19 as a social disease: Qualitative analysis of COVID-19 prevention needs, impact of control measures and community responses among racialized/ethnic minorities in Antwerp, Belgium. Int J Equity Health.

[8] Vilar-Compte M, Hernández-F M, Gaitán-Rossi P, Pérez V, Teruel G (2022). Associations of the COVID-19 pandemic with social well-being indicators in Mexico. International Journal for Equity in Health.

[9] Holst H, Fessler A, Niehoff S (2022). Covid-19, Ungleichheit und (Erwerbs-)Arbeit – zur Relevanz sozialer Klasse in der Pandemie (Covid-19, Inequality and (Paid) Work – On the Relevance of Social Class in the Pandemic). Z Soziol.

[10] Burström B, Tao W (2020). Social determinants of health and inequalities in COVID-19. Eur J Pub Health.

[11] Holden TM, Simon MA, Arnold DT, Halloway V, Gerardin J (2022). Structural racism and COVID-19 response: higher risk of exposure drives disparate COVID-19 deaths among Black and Hispanic/Latinx residents of Illinois, USA. BMC Public Health.

[12] Turner-Musa J, Ajayi O, Kemp L (2020). Examining social determinants of health, stigma, and COVID-19 disparities. Healthcare.

[13] World Health Organization. COVID-19 and the social determinants of health and health equity: Evidence brief. https://www.who.int/publications/i/item/9789240038387 (2021). Accessed 12 Sept 2023.

[14] Brookfield S (1998). Against naive romanticism: From celebration to the critical analysis of experience. Stud Contin Educ.

[15] Bourdieu P, Wacquant LJ (1992). An invitation to reflexive sociology.

[16] Costa C, Murphy M. Bourdieu and the application of habitus across the social sciences. In: Costa C, Murphy M, editors. Bourdieu, habitus and social research. Houndmills: Palgrave Macmillan; 2015. P. 3–20. 10.1057/9781137496928_1.

[17] Bourdieu P (2013). Outline of a theory of practice.

[18] Wacquant L, Stones R (1998). Pierre Bourdieu. Key sociological thinkers.

[19] Bourdieu P. The Forms of Capital, Handbook of theory and research for the sociology of education, In Richardson JG, editor. New York: Greenwood Press; 1986. p. 241–258.

[20] Grossman M (1972). On the concept of health capital and the demand for health. J Polit Econ.

[21] Shim JK (2010). Cultural health capital: A theoretical approach to understanding health care interactions and the dynamics of unequal treatment. J Health Soc Behav.

[22] Adams C, Harder BM, Chatterjee A, Hayes ML (2019). Healthworlds, cultural health toolkits, and choice: How acculturation affects patients’ views of prescription drugs and prescription drug advertising. Qual Health Res.

[23] Logan RG, Daley EM, Vamos CA, Louis-Jacques A, Marhefka SL (2021). “When is health care actually going to be care?” The lived experience of family planning care among young black women. Qual Health Res.

[24] Harsløf I, Larsen K, Bambra C (2023). When health is wealth: Occupationally differentiated patterns of health capital in post-industrial Europe. Soc Theory Health.

[25] Schneider-Kamp A (2021). Health capital: Toward a conceptual framework for understanding the construction of individual health. Soc Theory Health.

[26] Bohnsack R, Flick U (2014). Documentary method. The SAGE handbook of qualitative data analyses.

[27] Pfaff N, Bohnsack R, Weller W, Pfaff N, Bohnsack P, Weller W (2010). Reconstructive research and the documentary method in Brazilian and German educational science. Qualitative analysis and documentary method: International Educational Research.

[28] Glaser BG, Strauss AL. The discovery of grounded theory: Strategies for qualitative research. AldineTransaction - A Division of Transaction Publishers; 2006.

[29] Bohnsack R, Nohl AM (2003). Youth culture as practical innovation: Turkish German youth, time out'and the actionisms of breakdance. Eur J Cult Stud.

[30] Roshchina Y, Roshchin S, Rozhkova K (2022). Determinants of COVID-19 vaccine hesitancy and resistance in Russia. Vaccine.

[31] Schneider-Kamp A (2022). COVID-19 Vaccine Hesitancy in Denmark and Russia: A qualitative typology at the nexus of agency and health capital. SSM-Qualitative Research in Health.

[32] Mezirow J, Cranton P (1997). Transformative learning: theory to practice. New directions for adult and continuing education.

[33] Bourdieu P (2000). Pascalian meditations.

[34] Hidano A, Page B, Rudge JW, Enticott G (2022). Luck perception is associated with less frequent preventive practices and a higher number of social contacts among adults during the SARS-CoV-2 pandemic. Public Health in Practice.

[35] Bauman Z (2011). Collateral damage: Social inequalities in a global age.

[36] Sennett R (2006). The culture of new capitalism.

[37] Galasso V, Pons V, Profeta P, Becher M, Brouard S, Foucault M (2020). Gender differences in COVID-19 attitudes and behavior: Panel evidence from eight countries. Proc Natl Acad Sci.

[38] Broche-Pérez Y, Fernández-Fleites Z, Fernández-Castillo E, Jiménez-Puig E, Vizcaíno-Escobar AE, Ferrer-Lozano DM, Martín-González R (2021). "Anxiety, health self-perception, and worry about the resurgence of COVID-19 predict fear reactions among genders in the Cuban population.". Front Glob Women's Health.

[39] Chauhan P (2021). Gendering COVID-19: Impact of the pandemic on women’s burden of unpaid work in India. Gend Issues.

[40] Avery AR, Tsang S, Seto EY, Duncan GE (2021). Differences in stress and anxiety among women with and without children in the household during the early months of the COVID-19 pandemic. Front Public Health.

[41] Elanda Y (2021). The construction of an ideal mother amid the Covid 19 pandemic: Gender injustice experienced by career women while working from home. J Gender Stud.

